# Antagonistic Interactions of “Ya-Sa-Marn-Phlae” Ethanol Extract in Combination with Topical Antiseptics against Clinical Isolates of *Staphylococcus aureus*

**DOI:** 10.1155/2014/867603

**Published:** 2014-05-05

**Authors:** Sasitorn Chusri, Sirirat Tongrod, Julalak Chokpaisarn, Surasak Limsuwan, Supayang Piyawan Voravuthikunchai

**Affiliations:** ^1^Faculty of Traditional Thai Medicine, Prince of Songkla University, Hat Yai, Songkla 90110, Thailand; ^2^Natural Product Research Center of Excellence, Prince of Songkla University, Hat Yai, Songkla 90110, Thailand; ^3^Department of Microbiology, Faculty of Science, Prince of Songkla University, Hat Yai, Songkhla 90112, Thailand

## Abstract

This investigation was aimed at assessing a possible interaction of a traditional Thai herbal recipe, “Ya-Sa-Marn-Phlae (YSMP),” used for wound treatments with topical antiseptics, povidone-iodine (PI) solution and hydrogen peroxide (H_2_O_2_), and effects of THR-SK010 alone and the combinations on *Staphylococcus aureus*. Antibacterial activities of ethanol extracts from the herbal recipe were determined against both methicillin resistant *Staphylococcus aureus* (MRSA) and methicillin-susceptible *S. aureus* (MSSA). YSMP exhibited remarkable antistaphylococcal activity with MIC values of 3.9–7.8 **μ**g/mL. This recipe possessed bacteriostatic activity and did not reduce the tolerance of both MRSA and MSSA isolates to the high ionic strength. Interaction between THR-SK010 and the antiseptics was carried out by checkerboard testing and time-kill assay. Both indifferent and slightly antagonistic effects were observed with THR-SK010/PI and THR-SK010/H_2_O_2_ combinations against the tested isolates. In addition to commercially available antiseptics, THR-SK010 offered additional therapeutic options for the decolonization of MRSA and MSSA. Topical application of plant extracts with antioxidant activity, such as THR-SK010, should not be used immediately with PI or H_2_O_2_ and further investigation on this interaction is needed.

## 1. Introduction

Besides being one of the most important human pathogens,* Staphylococcus aureus* colonizes large proportions of human populations. Several researchers proposed that nasal cavities are considered to be the primary colonization site [1, 2], and 20% of the population is classified as persistent carrier while 60% is classified as intermittent carrier. Although the human throat is less well studied as a carriage site, there are some investigations reporting that the throat is the most common carriage site [3, 4]. Risk and outcome of nosocomial staphylococcal bacteraemia of* S. aureus* nasal carriers and noncarriers are significantly different and the bacteraemia is three times more frequent in the carriers than in noncarriers [5]. Approximately, 30–80% of staphylococcal infections are of endogenous origin in nasal carriers [2, 6]. Hence, effective disinfection and antisepsis are very essential in preventing infections, particularly within health care settings.

Decolonization agents such as chlorhexidine, mupirocin, and triclosan have been used to eradicate nasal and hand carriage of methicillin-susceptible* S. aureus* (MSSA) and MRSA. Unfortunately, emergence of resistant isolates as a result of long-term and intermittent usage of these decolonization agents has been frequently reported [7]. Increasing effort has been focused on utilizing secondary metabolites of medicinal plants which form the backbone of traditional medicine as alternative antiseptics [8, 9]. In addition,* in vitro* activities of some plant-derived compounds in combination with topical antiseptics against MRSA have been investigated for offering additional therapeutic options for the decolonization of this pathogen [10–12]. Although only little information is available for herbal recipes which are in current use by folk healers, some recipes from China [13], India [14], or Ghana [15] possessed interesting biological activities.

A traditional Thai herbal recipe, namely, “Ya-Sa-Marn-Phlae” (YSMP or THR-SK010) was obtained from a folk healer, Mr. Somporn Chanwanisakul, and has been used for the treatment of wounds and skin infections. The ethanol extract of this recipe exhibited low toxicity and possessed antibacterial, antibiofilm, anti-inflammatory, and antioxidant activities [16–19]. In this study, we further investigated the antibacterial efficacy of THR-SK010 in combination with topical antiseptics (povidone-iodine and hydrogen peroxide) against both MRSA from infective origins and MSSA from colonization origins in order to answer whether any different effects occur between the bacteria from different sources. Effects of THR-SK010 alone and the combinations on staphylococcal cell membrane functions were additionally observed in high ionic strength environment.

## 2. Material and Methods

### 2.1. Extraction of “Ya-Sa-Marn-Phlae”

Powdered YSMP (500 g) consists of equal amounts (125 g) of* Curcuma longa* L. (rhizome),* Areca catechu* L. (seed),* Oryza sativa* L. (seed), and* Garcinia mangostana* L. (pericarp). Plant parts were locally collected and reference voucher specimens were deposited at Faculty of Traditional Thai Medicine, Prince of Songkla University, Hat Yai, Songkhla, Thailand. The powder was macerated with 95% ethanol for seven days (1 : 5; w/v). After filtration through a Whatman number 1 filter paper, this filtrate was removed with a rotatory evaporator and kept at 55°C until it was completely dry. Yield of the ethanol extract that was calculated as the ratio of the weight of the extract to the weight of the crude herb powder was 6.45% (w/w). Samples were stored in a sterile screw-capped bottle at −20°C and dissolved in dimethylsulfoxide (DMSO; Merck, Germany) before use [17].

### 2.2. Tested Bacterial Strains

Five isolates of methicillin resistant* Staphylococcus aureus* (MRSA) and five isolates of methicillin-susceptible* Staphylococcus aureus* (MSSA) obtained from the Natural Products Research Center, Faculty of Science, Prince of Songkla University, were used throughout the study. Antibiotic susceptibility and molecular characteristics of the human isolates have been reported previously [20].

### 2.3. Determination of Antistaphylococcal Activities of the Herbal Recipe Extract and Topical Antiseptics

Overnight suspensions of tested staphylococcal isolates were prepared following inoculation of Muller Hinton broth (MHB; Becton, Dickinson, and Company, France) with three to five well-isolated colonies from TSA. The suspension was adjusted to a 0.5 McFarland standard (1.5 × 10^8^ CFU/mL) and then diluted in MHB to generate a final concentration of 1 × 10^6^ CFU/mL. A stock solution of the herbal recipe (100 mg/mL in DMSO) was diluted in MHB to produce a working solution of 2 mg/mL. Povidone-iodine (PI) solution which is an aqueous solution of 10% PI to 1% available iodine (Betadine HR, IDS Manufacturing Ltd., Thailand) and hydrogen peroxide (H_2_O_2_; Merck, Germany) were diluted in MHB to obtain working suspensions of 5% and 1% (v/v), respectively. To determine the MICs of the antibacterial agents, broth microdilution assays were performed in line with CLSI guidelines [21]. Each well contained 100 *μ*L of tested antimicrobial agents (the recipe extracts 1000–0.49 *μ*g/mL, PI 5–2.4 × 10^−3^ (%; v/v), and H_2_O_2_ 1–4.9 × 10^−4^ (%; v/v)) and 100 *μ*L of the bacterial suspension and incubated for 24 h at 37°C. DMSO at final concentrations of 1% (v/v) was employed as negative control solvent. The MIC was then determined as the lowest concentration showing no growth using optical density (OD) at 595 nm (OD_595 nm_) on a microplate reader (Sunrise; Tecan Group Ltd.). The assay was repeated in triplicate.

### 2.4. Effect of the Effective Herbal Recipe on Bacterial Halotolerance

Effect of sub-MIC (1/2xMIC), MIC, or supra-MIC (2xMIC, 4xMIC, and 8xMIC) of THR-SK010 extract on the growth of representative isolates,* S. aureus* ATCC 29213, MRSA NPRC R001, and MSSA NPRC S003 on TSA and TSA supplemented with 7.5% (w/v) sodium chloride (NaCl; Merck, Germany), was evaluated. Suspensions of the isolates prepared as described above (1.5 × 10^8^ CFU/mL; 1 mL) were incubated with 1 mL of each concentration of the ethanol extract for 24 h. An aliquot (10 *μ*L) of samples was removed and cultured onto TSA and TSA supplemented with 7.5% NaCl. The viable cells on TSA-NaCl and TSA were enumerated after incubation at 37°C for 48 h [22].

### 2.5. Interaction of the Effective Herbal Recipe and Antiseptics

To assess the synergistic or antagonistic activity of THR-SK010/PI and THR-SK010/H_2_O_2_ combinations, fraction inhibitory concentration (FIC) was determined by chequerboard assay. In brief, serial double 2-fold dilutions of the antimicrobial compounds were prepared (the recipe extracts 2000–0.49 *μ*g/mL, PI 5–2.4 × 10^−3^ (%; v/v), and H_2_O_2_ 1–4.9 × 10^−4^ (%; v/v)) (2,000 to 0.97 *μ*g/mL for THR-SK010, 10 to 4.9 × 10^−3^ (%; v/v) for PI, and 2 to 9.8 × 10^−4^ (%; v/v) for H_2_O_2_). Fifty microlitres of each antiseptic was added to the rows of a 96-well microtiter plate in decreasing concentrations and 50 *μ*L of the recipe extract was added to the columns in reducing concentrations. The wells were then inoculated with 100 *μ*L of the bacterial suspension containing 1 × 10^6^ CFU/mL and the MIC of each agent alone and in combination was determined.

### 2.6. Time-Kill Assay

Interaction of THR-SK010 in combination with the antiseptics against representative isolates, MRSA NPRC R001, MSSA NPRC S001, and* S. aureus* ATCC 29213, was additionally conducted by time-kill methodology. An aliquot of bacterial suspensions prepared as described above (1 × 10^6^ CFU/mL; 1 mL) was incubated with 1 mL of the antibacterial agents alone, was incubated at 37°C with 1 mL of the antibacterial agents alone, the combination of THR-SK010 and PI or the combination of THR-SK010 and H_2_O_2_. A sample of 100 *μ*L was taken every 4 h up to 24 h and subsequently measured for the bacterial growth by reading the OD_595 nm_.

## 3. Results and Discussion

The MICs of H_2_O_2_, PI, and THR-SK010 extract for MRSA and MSSA isolates were 0.31–0.63, 2 × 10^−3^–8 × 10^−3^, and 3.9–7.8 *μ*g/mL, respectively. Bactericidal activity and effect of THR-SK010 extract on bacterial halotolerance were further carried out with representative isolates, MRSA NPRC R001, MSSA NPRC S003, and* S. aureus* ATCC 29213 (data not shown). Patterns of cell survival after treatment were similar among different isolates. Numbers of viable cells of the isolates after exposure to 1/2xMIC, 1xMIC, 2xMIC, and 4xMIC of the extract for 24 h decreased 2-3 log folds. The level of tested isolates was reduced by at least 5 log folds after treatment with 8xMIC of the extract for 24 h. For TSA+7.5% NaCl, the numbers of viable cells after treatment with the extract were similar to that of TSA. This indicated that treatment with THR-SK010 extract did not affect the staphylococcal halotolerance. Osmotolerance of* S. aureus* to NaCl is described as its ability to maintain the structural integrity of its cytoplasmic membrane. Earlier investigations indicated that sublethal injury of microbial cell membrane caused by plant extract and plant-derived compounds may alter their permeability and affect the ability of the membrane to osmoregulate the cell adequately [22–24]. Therefore, the antistaphylococcal mechanisms of the extract may not cause any alteration to the staphylococcal cell membrane function.

Using ΣFIC values for synergy as ≤0.5 and antagonism as >4.0 as recommended by the American Society for Microbiology (Table 1), no synergistic interactions were observed between combinations of THR-SK010/PI and THR-SK010/H_2_O_2_ when tested against staphylococcal isolates. However, a tendency for an indifferent antibacterial effect was observed with THR-SK010/PI combination against MSSA isolates and THR-SK010/H_2_O_2_ against both MRSA and MSSA isolates and THR-SK010/PI combination against MRSA isolates which exhibited an antagonistic antibacterial effect.

**Figure 1:**
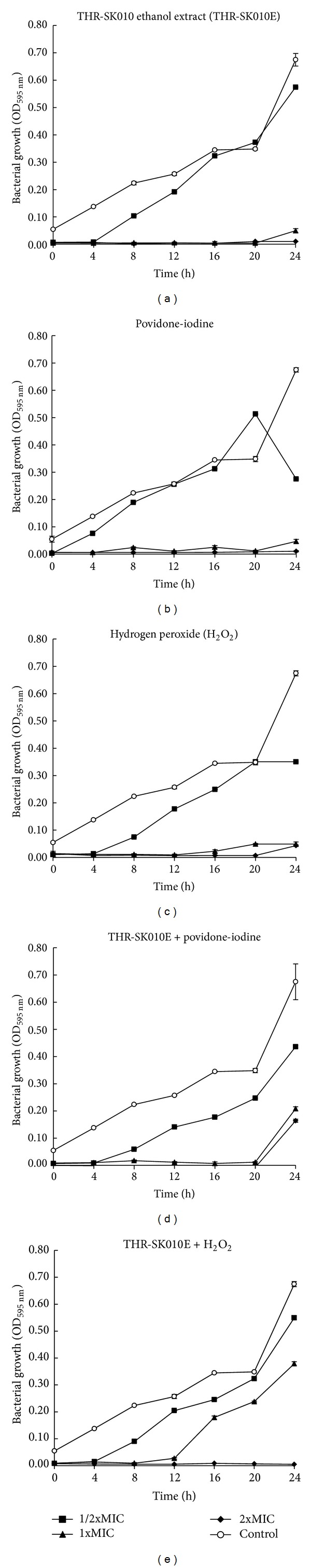
Bacterial growth inhibition patterns of THR-SK010 ethanol extract (a), povidone-iodine (PI; (b)), and hydrogen peroxide (H_2_O_2_; (c)) and combinations of THR-SK010/PI (d) and THR-SK010/H_2_O_2_ (e) against methicillin resistant* Staphylococcus aureus* NPRC R001. Minimum inhibitory concentration (MIC) values of THR-SK010E, H_2_O_2,_ and PI were 3.9, 4 × 10^−3^, and 0.63 *μ*g/mL, respectively.

The combinations were further evaluated in bacterial growth inhibition patterns using a time-kill method as previously described with a slight modification (Figure 1). THR-SK010 extract, PI, and H_2_O_2_ at 1xMIC and 2xMIC demonstrated significant* in vitro* bacterial growth inhibition for 24 h. Antagonism was noted between the recipe and PI combination. Although bacterial growth inhibitions of this combination against MRSA, MSSA, and* S. aureu*s ATCC 29213 were observed, a regrowth of these isolates within 20 h was detected. Moreover, treatment with 1xMIC of the combination of the recipe and H_2_O_2_ caused regrowth of the isolates after 12 h.

This investigation shows for the first time (to our knowledge) that the Thai herbal recipe may not work effectively in combination with topical antiseptics as shown by their antagonistic effects against multidrug resistant bacteria. Commercially available antiseptics, PI and H_2_O_2_, were chosen for their remarkable antistaphylococcal activity against both planktonic and biofilm growth modes [25–27] and their rapid action against bacteria [28]. According to the FIC indices in the present study, the combination of THR-SK010 and H_2_O_2_ or THR-SK010 and PI exerted indifferent antibacterial activity against most of the tested isolates. Observation of the bacterial growth inhibition patterns in the presence of each antibacterial agent and in the presence of the combinations was additionally performed. Slightly antagonistic effects were detected in these combinations. It is well established that the antibacterial activity of PI is due to the slow release of iodine, which shows a potent oxidative activity. Similarly, H_2_O_2_ is thought to kill bacteria by rapidly damaging DNA with highly reactive hydroxyl radicals [28]. Therefore, compounds with antioxidant activity are expected to reduce the efficacy or inhibit the activity of PI and H_2_O_2_. Interferences of the biological activities of the antiseptics including their antibacterial activity have been found from both chemicals such as sodium thiosulfate [29] and plant-derived compounds such as quercetin [30] and catechins [31]. The herbal components of THR-SK010, curcuminoids from* Curcuma longa* [32], and xanthones from* Garcinia mangostana* [33] possessed strong antioxidant activities; thus, the biological activity might have an inhibitory effect on the antibacterial activity of PI and H_2_O_2_.

## 4. **Conclusion**

In summary, our data revealed that traditional Thai herbal recipe THR-SK010 possessed notable antibacterial activity against both MRSA and MSSA. The recipe exhibited bacteriostatic activity and did not affect the halotolerance of the pathogen. Our findings indicate that the topical recipe reduces the antibacterial activity of both PI and H_2_O_2_ which is probably due to the antioxidant activity of its herbal components.

## Figures and Tables

**Table 1:** Fractional inhibitory concentration indices (FIC) of antimicrobial agents determined by a two-dimensional chequerboard assay against methicillin resistant *Staphylococcus aureus* (MRSA), methicillin-susceptible *Staphylococcus aureus* (MSSA), and *S. aureus* ATCC 29213^a^ when combined with an effective herbal formula, THR-SK010E, and topical antiseptics (povidone-iodine; PI and hydrogen peroxide; H_2_O_2_).

Antibacterial agents	ΣFIC ranges	Percentage of the tested isolated		
		Synergy	Indifference	Antagonism
		ΣFIC ≤ 0.5	0.5 < ΣFIC ≤ 4	ΣFIC > 4
THR-SK010/PI				
MRSA (*n* = 5)	5.00–9.00			100
MSSA (*n* = 5)	2.00–5.00		80	20
THR-SK010/H_2_O_2_				
MRSA (*n* = 5)	1.05–2.53		100	
MSSA (*n* = 5)	0.51–2.14		100	

^a^ΣFIC indices of pared combinations of THR-SK010 and povidone-iodine and THR-SK010 and H_2_O_2_ against *S. aureus* ATCC 29213 were 3.00 and 1.06, respectively.
